# Influence of Polycaprolactone Concentration and Solvent Type on the Dimensions and Morphology of Electrosprayed Particles

**DOI:** 10.3390/ma16052122

**Published:** 2023-03-06

**Authors:** Laura Alberto, Lohitha Kalluri, Jing Qu, Yongfeng Zhao, Yuanyuan Duan

**Affiliations:** 1 Department of Biomedical Materials Science, University of Mississippi Medical Center, Jackson, MS 39216, USA; 2 Department of Chemistry, Physics and Atmospheric Sciences, Jackson State University, Jackson, MS 39216, USA

**Keywords:** dentistry, electrospray, microparticles, morphology, PCL, solvent

## Abstract

Polycaprolactone (PCL) micro- and nanoparticles produced using the electrospraying technique present high drug encapsulation capacity, a controllable surface area, and a good cost–benefit ratio. PCL is also considered a non-toxic polymeric material with excellent biocompatibility and biodegradability. All these characteristics make PCL micro- and nanoparticles a promising material for tissue engineering regeneration, drug delivery, and surface modification in dentistry. In this study, PCL electrosprayed specimens were produced and analyzed to determine their morphology and size. Three PCL concentrations (2, 4, and 6 wt%) and three solvent types (chloroform (CF), dimethylformamide (DMF), and acetic acid (AA)) with various solvent mixtures ratios (1:1 CF/DMF, 3:1 CF/DMF, 100% CF, 1:1 AA/CF, 3:1 AA/CF, and 100% AA) were used while keeping the remaining electrospray parameters constant. SEM images followed by ImageJ analysis showed a change in the morphology and size of the particles among various tested groups. A two-way ANOVA demonstrated a statistically significant interaction (*p* < 0.001) between PCL concentration and solvents on the size of the particles. With the increase in the PCL concentration, an increase in the number of fibers was observed among all the groups. The morphology and dimensions of the electrosprayed particles, as well as the presence of fibers, were significantly dependent on the PCL concentration, choice of solvent, and solvent ratio.

## 1. Introduction

Micro- and nanoparticles were used in biomedical science as drug delivery systems, tissue engineering, biosensors, and control release systems [1]. Such technology also has promising potential in dental diagnosis and therapeutics [2]. Different polymers were used to produce particles with specific size ranges and porous distributions to encapsulate or chemically bond with a range of drugs and biomolecules according to the intended application. Polycaprolactone (PCL) is considered an environmentally green, non-toxic material with appropriate mechanical properties, excellent biocompatibility, and biodegradability, with a degradation time of up to 2 years, with minimum acidic byproducts [3,4,5,6,7]. In addition to these material advantages, PCL micro- and nanoparticles produced via the electrospraying technique present high encapsulation capacity, a controllable surface area, high porosity, and a good cost–benefit ratio [8,9]. 

Electrospraying is an emerging process due to the fact that it can be carried out at room temperature, preventing the degradation of proteins and polymers [4,10]. The basic configuration of an electrospray system consists of a pump where a syringe containing a solution is placed with a suitable metal capillary. A high-voltage supply is attached to the needle and a constant flow rate is applied. This process induces the formation of a positively charged stable Taylor cone and the consequently charged droplets. With solvent evaporation, shrinkage of the droplets occurs. The approximation of the similar charges produces a coulombic explosion that reduces and propels the newly generated particles until their deposition on an electrically grounded collector [4,8,11,12,13]. 

Many factors play key roles in the final morphology, size, porosity, and distribution of the generated electrosprayed particles. Changes in the operating parameters (flow rate, applied voltage, and spinneret–collector distance), as well as in the solution parameters, such as solvent selection (binary and lone solvents) and polymer concentration, allow the operator to control the electrospray particles for the desired outcomes and applications [4,5,7,8]. In this study, solution parameters were investigated due to their primary importance to tailor the morphological characteristics and size of electrospray particles. 

The physical properties of the solutions (viscosity, surface tension, electrical conductivity, and density) influence the Taylor cone formation, charged droplet generation, particle morphology, particle size, and presence of fibers in the resulting electrospray particles. They are dependent on the polymer’s molecular weight and concentration, as well as the type, ratio, and solubility of the solvent [4,8,14]. Some relationships related to the physical properties of solutions and the resultant outcome are known. For example, the higher the molecular weight and concentration of the polymer, the higher the entanglement of the polymer chains, viscosity, and tendency to form fibers. Thus, to generate particles, a low chain entanglement and low solution viscosity are required. When it comes to the formation of a stable Taylor cone (which is essential for micro- and nanoparticle production), the electrical stress must overcome the surface tension of the polymeric solution [7,15]. 

The solvent solubility parameters and evaporation rate are also highly important for the morphology of the electrosprayed particles and how the results differ among the samples produced. Chloroform (CF) was used as the main solvent for PCL due to its excellent solubility parameters [4,5]. Due to the rapid chloroform evaporation, the particles produced with this solvent usually present small pores [16]. However, concerns about this solvent’s toxicity and carcinogenicity have limited its use in the biomedical field. Acetic acid (AA) has good solubility parameters for PCL and is considered a green solvent [17,18]. The acceptable daily intakes of acetic acid have not been established yet, although intake estimations vary from 1 to 2.1 g/day for individuals over two years old [19]. Dimethylformamide (DMF) has partial solubility parameters for PCL; therefore, the use of this substance as a co-solvent can modify the chain–solvent interactions, as well as the chain entanglement and particle morphology [4]. This solvent has hepatotoxic potential, although its toxicity is dependent on the dosage and time of exposure, as well as the patient’s previous liver condition [20]. The use of binary or ternary solvent systems to dissolve PCL is done to adjust the structure and morphology of the particles produced via electrospraying. Toxicity and environmental properties can also be improved by solvent mixtures [4]. 

Potential drug delivery applications of micro- and nanoparticles in the oral cavity include the treatment of inflammatory reactions and infections [21], periodontal disease [22,23], seeded scaffolds for bone regeneration [10], synthetic graft material [9], and surface modification of implants with osteogenic coatings [24], which can be obtained by using, among other techniques, an electrospraying process or one-step microsphere-nanofibers production with the same apparatus [25,26]. The high surface-area-to-volume ratio of the electrosprayed particles benefits the delivery of the active ingredients [5], once it increases the release rate, due to the faster fluids penetration and polymer degradation [10]. The objective of this study was to analyze the influence of solvent selection and PCL concentration on the dimension and morphology of the final product of the electrospraying process of PCL. The elaborated hypothesis was that solutions formulated with lower PCL concentration and solvents with better solubility would produce significantly smaller particles with a rounder morphology.

## 2. Materials and Methods

The solution components PCL (Mw = 80,000 g mol^−1^), chloroform (≥99.5%), N,N-Dimethylformamide (99.8%), and glacial acetic acid (≥99%) were purchased and used as received from Sigma-Aldrich (Millipore Sigma, Saint Louis, MO, USA). Distilled water was used for solution preparation for the experimental procedures.

In this study, the morphology and size of the electrosprayed particles were evaluated at three PCL concentrations (2 wt%, 4 wt%, and 6 wt%) using six different solvent mixtures (1:1 CF/DMF, 3:1 CF/DMF, 100% CF, 1:1 AA/CF, 3:1 AA/CF, and 100% AA), divided into two groups (CF/DMF and AA/CF). Thus, a total of eighteen groups of specimens with varying PCL and solvent concentrations containing three specimens (n = 3) per group were prepared, as tabulated in Table 1.

Homogeneous dissolution of the PCL pellets was accomplished using a bench rocker at room temperature. The solutions were transferred to 10 mL syringes attached to stainless steel 16 gauge needles (4 inches in length) and placed in an electrospraying pump (Inovenso Inc., Boston, MA, USA). Afterward, the electrode of a power supply was applied to the needle. Constant electrospraying parameters, such as an applied voltage of 15 kV, a spinneret–collector distance of 12 cm, and a flow rate of 0.7 mL/h, were used to produce all samples [3,13]. The polymer solutions were extruded from a syringe pump system toward the grounded collector for 5 min in laboratory conditions (23 ± 2 °C temperature and 50 ± 1% relative humidity). The grounded plate was covered using aluminum foil for specimen collection.

Due to the non-conductive nature of the polymeric particles, the samples were sputter-coated (Leica Microsystems, Vienna, Austria) with an 8.0 nm Au-Pd coating. The particles’ morphology was assessed using a scanning electron microscope (SEM, Carl Zeiss, Oberkochen, Germany). The quantitative evaluation of particle size was done by measuring the diameters of 30 random particles per sample (n = 54) from the obtained SEM micrographs using ImageJ software (National Institutes of Health, Bethesda, MD, USA) [5,9,13,24]. To estimate the influence of the PCL concentration and solvent on the size of the electrosprayed particles, statistical analysis was performed using two-way ANOVA followed by Tukey’s test for pairwise comparisons (SigmaPlot 14, Systat Software, Chicago, IL, USA). This statistical analysis was chosen to analyze the effects of the independent variables (PCL concentration and solvent ratio) on the expected outcome (in this case, the mean size of the particles), along with the relationship and interactions between them with the outcome itself. Tukey’s test was used as the post hoc test to compare the difference between the mean of all possible pairs of all groups using a studentized range distribution once an equal number of subjects were observed in each group [27]. The hydrodynamic diameter (HD) of polymeric particles was determined via dynamic light scattering (DLS) using a Litesizer 100 from Anton Paar (Ashland, VA, USA). A small piece of 2 by 2 cm^2^ aluminum foil of each PCL sample that exclusively produced particles (2 wt% 100% CF, 2 wt% 1:1 AA/CF, 2 wt% 3:1 AA/CF, 2 wt% 100% AA, and 4 wt% 100% AA) was placed into 5.0 mL of distilled water. Then, the samples were sonicated for 10 min in order to transfer and dilute the PCL particles in water. Then, 2.0 mL of each resulting solution was placed in a disposable cuvette and measured three times at ambient temperature (25 °C). Additionally, the zeta potentials of the same samples were acquired to determine their surface charges using a Zetasizer Nano ZS instrument (Malvern Instruments Ltd., Malvern, UK).

## 3. Results

**Figure 1:**
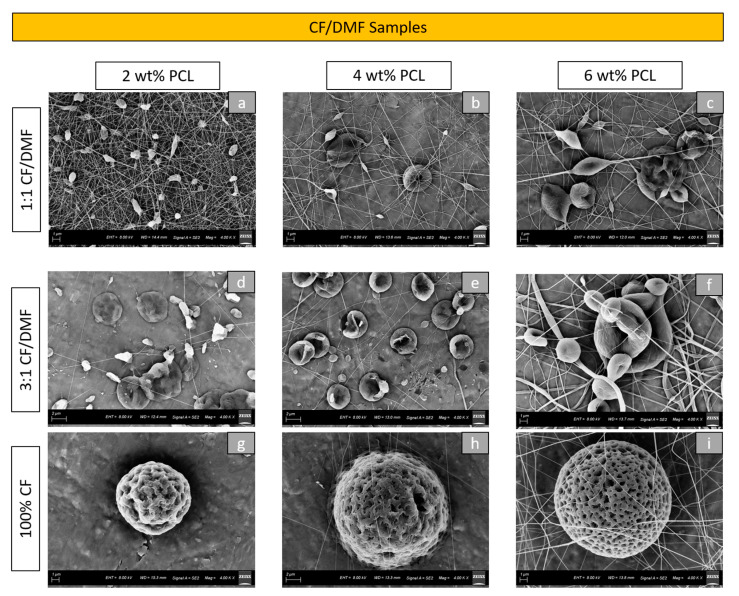
High magnification (4.00 kx) SEM images of PCL particles created by electrospraying: (**a**) 2 wt% PCL with 1:1 CF/DMF; (**b**) 4 wt% PCL with 1:1 CF/DMF; (**c**) 6 wt% PCL with 1:1 CF/DMF; (**d**) 2 wt% PCL with 3:1 CF/DMF; (**e**) 4 wt% PCL with 3:1 CF/DMF; (**f**) 6 wt% PCL with 3:1 CF/DMF; (**g**) 2 wt% PCL with 100% CF; (**h**) 4 wt% PCL with 100% CF; (**i**) 6 wt% PCL with 100% CF.

SEM images showed changes in the morphologies and sizes of the particles among various tested groups. The morphology of the particles formed in the CF/DMF group using 2 wt%, 4 wt%, and 6 wt% PCL concentrations was depicted in Figure 1. The choice and the ratio of solvents influenced the morphologies of the particles. An increase in CF concentration produced more rounded and textured PCL electrosprayed particles. Within the 100% CF solvent, the samples with 2 wt% PCL concentration demonstrated relatively small (mean particle diameter = 9.90 µm) and wrinkled particles, while the samples with 6 wt% PCL showed bigger particles (mean particle diameter = 11.35 µm) with high porosity (Figure 1g,i). The use of DMF increased the mixing time to obtain homogeneous mixtures (2:30 h for 1:1 CF/DMF vs. 2 h for other solutions in the group). Considering the morphology of the particles, higher DMF concentrations produced smoother surface particles and increased the number of fibers. The PCL concentration also impacted the morphology of the particles produced. With the increase in the PCL concentration, an increase in the number of fibers was observed among all the solvent combinations for this group (except for the 2 wt% 1:1 CF/DMF group).

**Figure 2:**
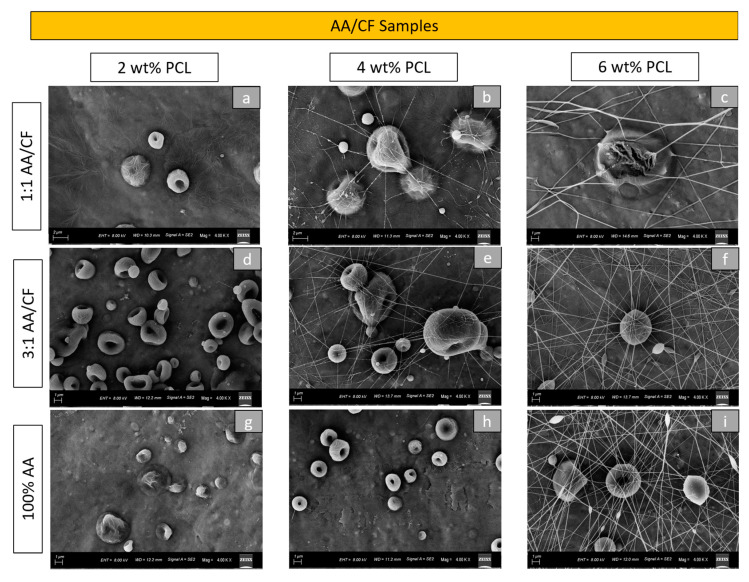
Morphological aspect of the electrosprayed particles produced under SEM (4.00 kx magnification): (**a**) 2 wt% PCL with 1:1 AA/CF; (**b**) 4 wt% PCL with 1:1 AA/CF; (**c**) 6 wt% PCL with 1:1 AA/CF; (**d**) 2 wt% PCL with 3:1 AA/CF; (**e**) 4 wt% PCL with 3:1 AA/CF; (**f**) 6 wt% PCL with 3:1 AA/CF; (**g**) 2 wt% PCL with 100% AA; (**h**) 4 wt% PCL 100% with AA; (**i**) 6 wt% PCL with 100% AA.

Figure 2 demonstrates the morphological aspect of the electrosprayed particles produced from 2 wt%, 4 wt%, and 6 wt% PCL using 1:1 AA/CF, 3:1 AA/CF, and 100% AA solvent concentrations under SEM. As in the previous group, the PCL concentration influenced the morphologies of the particles and the presence of fibers. It was clear that an increase in PCL concentration increased the number of fibers present among all groups. Furthermore, lower PCL concentrations induced a dimple-like morphology in the particles (Figure 2a,d,g) in this group. The use of 100% AA solvent required four hours to obtain homogenous solutions, while the remaining groups (with CF in the composition) required two hours for the same outcome. The use of AA as the sole solvent reduced the formation of fiber, even with a 4 wt% PCL concentration (Figure 2h).

The mean diameters of the particles for all groups measured using SEM micrographs and ImageJ software are depicted in Table 2. As demonstrated in the aforementioned table, and further in the two-way ANOVA analysis, an increase in the CF concentration within the same PCL concentration led to a statistically significant (*p* < 0.001) increase in particle size in both groups. A quantitative increase (but not statistically relevant for all groups) occurred as well due to an increase in the PCL concentration within the same solvent ratios. 

**Figure 3:**
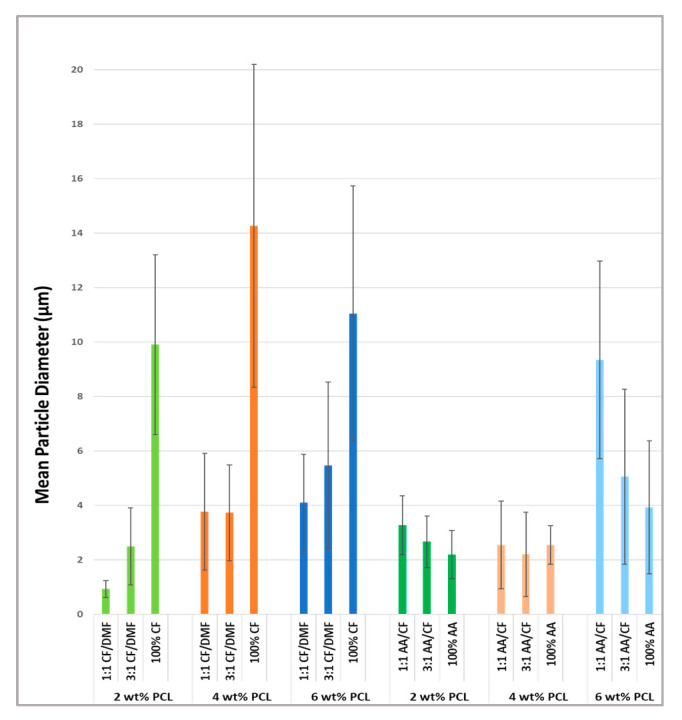
Mean particle diameters (in µm) and respective standard deviations for all the groups tested based on the SEM micrographs.

The two-way ANOVA demonstrated a statistically significant interaction (*p* < 0.001) between the PCL concentration and solvents on the size of the particles for the CF/DMF and AA/CF groups. Among the PCL concentrations and solvent mixtures tested, pairwise comparisons using Tukey’s test showed a statistically significant variation (*p* < 0.001) in the mean particle diameter of the particles between all the PCL concentration levels and solvent groups, except between 4 wt% PCL and 6 wt% PCL in the CF/DMF group (*p* = 0.549) and between 2 wt% PCL and 4 wt% PCL in the AA/CF group. Pairwise comparisons using Tukey’s test also showed a statistically significant variation (*p* < 0.050) in the mean particle diameter between all nine combinations of PCL and solvent concentrations in the CF/DMF group, except between the samples produced with the following solution composition: 4 wt% PCL with 1:1 CF/DMF vs. 4 wt% PCL with 3:1 CF/DMF (*p* = 0.994) and 6 wt% PCL with 1:1 CF/DMF vs. 4 wt% PCL with 1:1 CF/DMF (*p* = 0.757). For the AA/CF group, the exceptions for statistically significant variation (*p* < 0.001) in the pairwise comparisons using Tukey’s test were for the groups using 2 wt% PCL groups with 1:1 AA/CF vs. 3:1 AA/CF (*p* = 0.114) and 3:1 AA/CF vs. 100% AA (*p* = 0.267), all the solvent ratio combinations using 4% PCL (*p* between 0.494 and 1.00), 2 wt% vs. 4 wt% PCL concentration within 3:1 AA/CF (*p* = 0.290), and 2 wt% vs. 4 wt% PCL concentration within the 100% AA group (*p* = 0.465). A graph demonstrating the mean particle diameter in µm and the respective standard deviations for all the groups tested can be seen in Figure 3. 

**Figure 4:**
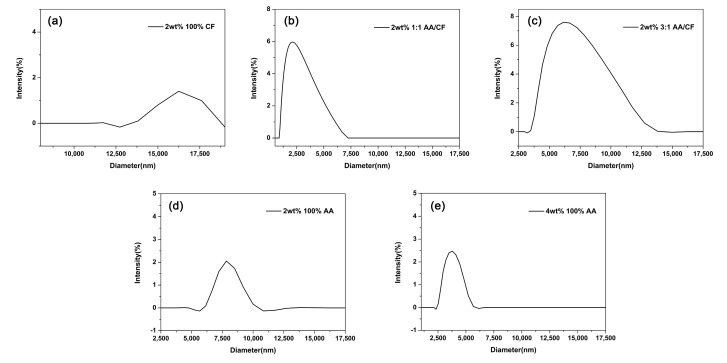
Dynamic light scattering (DLS) size distribution by intensity (%) of (**a**) 2 wt% 100% CF, (**b**) 2 wt% 1:1 AA/CF, (**c**) 2 wt% 3:1 AA/CF, (**d**) 2 wt% 100% AA, and (**e**) 4 wt% 100% AA, in nm.

DLS measurements of hydrodynamic size by intensity (%) followed by their standard deviations for particles produced with compositions of 2 wt% 100% CF, 2 wt% 1:1 AA/CF, 2 wt% 3:1 AA/CF, 2 wt% 100% AA, and 4 wt% 100% AA were 16.2 ± 0.0, 2.6 ± 1.0, 6.9 ± 1.8, 7.9 ± 0.5, and 3.8 ± 0.6 µm respectively, as exhibited in Figure 4 and Table 3. The polydispersity index (PDI), which is a measure of a sample’s heterogeneity based on the size, of all samples tested was between 0.05 and 0.5, as shown in Table 3. 

**Figure 5:**
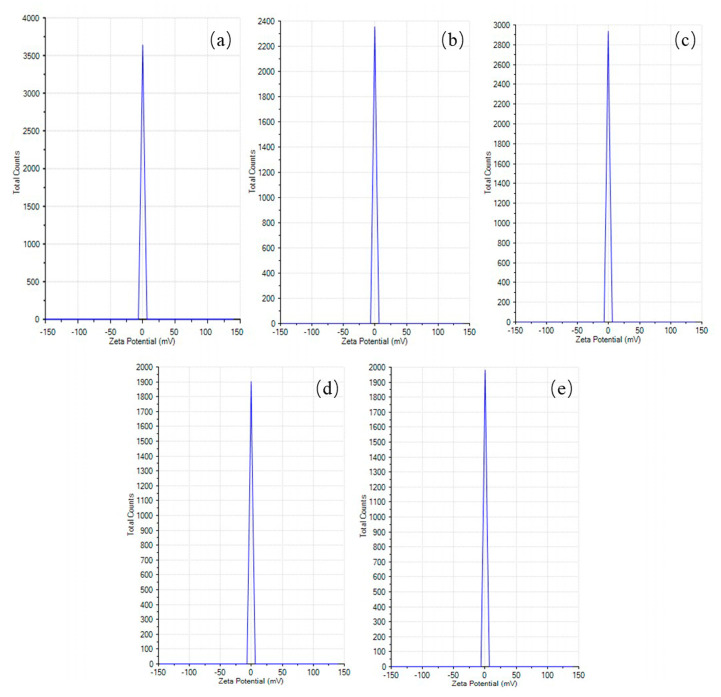
Zeta potential measurement for synthesized PCL particles: (**a**) 2 wt% 100% CF, (**b**) 2 wt% 1:1 AA/CF, (**c**) 2 wt% 3:1 AA/CF, (**d**) 2 wt% 100% AA, and (**e**) 4 wt% 100% AA.

The measurement of the electrical charge around the particles (zeta potential) is necessary for biomedical applications [28]. Figure 5 and Table 3 depict the zeta potential and its standard deviation of the PCL particles formulated using 2 wt% 100% CF, 2 wt% 1:1 AA/CF, 2 wt% 3:1 AA/CF, 2 wt% 100% AA, and 4 wt% 100% AA (−0.06 ± 2.32 mV, −0.51 ± 2.31 mV, −0.79 ± 2.31 mV, −0.49 ± 2.32 mV, and 0.19 ± 2.32 mV, respectively).

## 4. Discussion

High-molecular-weight PCL (Mw = 80,000 g mol^−1^) was used at 2 wt%, 4 wt%, and 6 wt% concentrations to produce electrosprayed specimens. Usually, high molecular weights are responsible for producing a spherical morphology via the precipitation of the entangled chains after solvent evaporation [4]. In the current study, the production of microparticles absent of fibers was achieved using the following solutions: 2 wt% PCL with 100% CF; 2, 4, and 6 wt% PCL with 100% AA; and 4 wt% PCL with 100% AA. Zang et al. [4] used PCL with molecular weights ranging from 70,000 to 90,000 g mol^−1^ dissolved in CF in their experiments. They reported the inability to produce particles even at very low PCL concentrations (0.5 wt%), in which only fibers were observed. Wang and collaborators [18] prepared a solution for the electrospray technique using a 10% PCL concentration and AA as the only solvent. In this study, round and smooth microspheres with the presence of fine fibers were observed. The authors related the presence of fibers to the high polymer concentration and consequently to the high entanglement of the polymer chains. A high polymer concentration is also associated with an increase in polymer solution viscosity. Taking this into consideration, solutions with low viscosity are easily broken into droplets by the applied voltage, resulting in particles, while high-viscosity solutions are more likely to produce fibers [29]. Similar outcomes were found in the present study. 

In this study, round particle morphology was only acquired in particles produced using 100% CF as the solvent. However, the use of such a solvent produced a significant increase in particle size compared with the remaining groups, a fact that was also directly proportional to the increase in PCL concentration. The results obtained in our study agree with the ones observed by Malagón-Romero et al. [30]. After testing a few parameters using PCL (Mn = 80,000) solutions at 7 wt% and 9 wt% using CF as the sole solvent, they concluded that increasing the PCL concentration led to an increase in the diameter of the spherical particles. Bock et al. [31] also demonstrated the spherical morphology of particles in high PCL concentrations (9 to 10 wt%). 

Solvent characteristics, such as solubility and evaporation rate, also influence the morphologies of the particles generated via electrospraying [29]. Solvents with good solubility for PCL, such as AA and CF, infiltrate between the polymer chains, which reduces the intermolecular entanglements of the polymer chains and facilitates the burst of the charged droplets into nano- and microparticles. In this scenario, within the particles, higher entanglement and condensed PCL chains tend to produce smaller and smoother particles, while low entanglement of the PCL chains produces a porous or wrinkled morphology. On the other hand, DMF presents partial solubility for PCL; therefore, it is usually used to produce fibers in the electrospinning process [4,31]. These observations are in accordance with the findings of our study. Particles produced exclusively with CF were round and textured, while particles produced with AA as the only solvent were smooth and smaller, indicating low and high entanglement of PCL chains within the particles, respectively. Another fact observed was that an increase in DMF proportion in the binary solvent systems of the CF/DMF samples increased the number of fibers, even in low PCL concentrations. However, when the two solvents presenting good solubility for PCL (CF and AA) were combined, fine fibers were observed in the sample with 4 wt% and 6 wt% PCL concentrations, probably due to the influence of CF (which produced fibers in the 4 and 6 wt% samples when it was the sole solvent). Fiber formation between particles was also observed in another study [31] when elevated concentrations of PCL were diluted in CF.

Solvent volatility is another important property in the electrospraying process since it has a direct impact on the size and morphology of the particles generated. Volatility is also related and inversely proportional to the boiling point of the solvent, which means that solvents with high boiling temperatures need high temperatures to evaporate and, therefore, are less volatile [10,12,32]. Solvents with high boiling temperatures, such as DMF (146 °C) [12] and AA (118.1 °C) [33], evaporate slowly, producing smaller particle sizes and smoother surfaces [10,32]. Similar characteristics were observed in samples produced in our and other studies using a binary solvent system containing DMF [12] and AA [18]. When solvents with high boiling temperatures are mixed with solvents with low boiling points, such as in the case of an AA/CF solution, the resulting mixture has its boiling point reduced. Campbel and Gieskes [33] analyzed acetic acid–chloroform systems in which the boiling point of AA was reduced from 118.1 °C to 81.5 °C when AA represented 57% of the mixture. This fact can explain the smooth but hollow cup-shaped morphology of the particles produced in the present study. Meanwhile, solvents with low boiling points, such as CF (61 °C), produce texturized larger particles [31]. The fast solvent evaporation prevents the polymer chain contraction and re-arrangement, resulting in porous or irregular particles [10,16,34]. In the present study, porous particles were observed in the samples produced with 100% CF. The number of pores on the particles’ surfaces seemed to increase with the PCL concentration in the samples produced with 100% CF, which was the inverse relationship found in a previous study [4].

Small, smooth, and free-of-fibers particles were produced in the current study using 2 wt% PCL with 1:1 AA/CF (3.26 µmmean size), 2 wt% PCL with 3:1 AA/CF (2.65 µm mean size), 2 wt% PCL with 100% AA (2.18 µm mean size), and 4 wt% PCL 100% with AA (2.54 µm mean size). The particles produced with 2 wt% PCL with a 100% CF solution were wrinkled and free of fibers but presented higher mean diameters (9.9 µm) compared with the aforementioned samples. Small and wrinkled particles present a high surface area to volume ratio and, consequently, faster fluids penetration, polymer degradation, and release rate. As a consequence of these characteristics, such particles are preferred for the delivery of active ingredients [10]. For dental applications, PCL particles were produced via the nanoprecipitation method to encapsulate substances such as curcumin and provide antimicrobial activity against periodontal pathogens on a multispecies biofilm when activated by blue light, showing no cytotoxicity toward the surrounding tissues [35]. Melatonin was also incorporated into PCL to develop a new synthetic graft material due to the inhibitory properties on osteoclast formation and osteoclastic activity of this hormone [9]. For medical applications, PCL particles were used to encapsulate hormones, such as β-estradiol (a contraceptive and hypocholesterolemic drug); anti-inflammatory drugs, such as budesonide (to control and prevent symptoms caused by asthma); and paclitaxel (a chemotherapy drug) [10]. 

In the DLS analysis, polydispersity index (PDI) values < 0.05 are more likely in monodisperse samples, while values >0.7 are considered to denote a broad size range (polydisperse) in the distribution of particles [36]. Based on this, the particles produced with the 2 wt% 1:1 AA/CF sample were considered to be close to monodisperse (PDI = 0.05), while the remaining samples analyzed indicated good uniformity and homogeneity in terms of the particle size and distribution (PDI ranging from 0.3 to 0.5).

Large positive and negative zeta potential values (±30 mV) usually exhibit high stability and, consequently, low aggregation of the particles in the suspension due to the strong electrostatic repulsion between them [28,37]. In the current study, the average values for the zeta potential were found in a range from −0.79 mV to 0.19 mV, suggesting a high flocculation rate. To the best of our knowledge, a consolidated protocol has not yet been developed to analyze the hydrodynamic size and zeta potential of micro- and nanoparticles produced via an electrospray technique; therefore, this study could serve as a guideline for future studies. The PCL particles prepared via double emulsification also presented a zeta potential close to the neutral charge [38], which agrees with the finds of the present study. Therefore, particle size and method of preparation can affect the magnitude of the zeta potential. A zeta potential of −16.8 mV was reported for PCL nanoparticles produced via the nano-precipitation technique [28], with an average particle size of 200 nm. 

Future studies focused on the degradation properties, and cytotoxicity can determine the applicability of the samples produced for biomedical applications, especially in the dental field. With the parameters tuned, the encapsulation of drugs and biological molecules, such as tetracycline, triclosan, bone morphogenetic proteins (BMPs), and hydroxyapatite, can be tested to provide treatment of oral inflammatory reactions and infections and enhance bone regeneration. 

## 5. Conclusions

The production of PCL nano- and microparticles via the electrospraying technique is a versatile process. In this study, it was observed that the solvent used, as well as the PCL concentration, played a major role in the morphology and size of the electrosprayed particles. However, the hypothesis elaborated was partially rejected since the use of CF as the exclusive solvent, even in the lowest PCL concentration solution, produced particles with large diameters compared with the remaining groups.

## Figures and Tables

**Table 1:** Overall study design demonstrating the test groups and samples analyzed.

PCL Concentration	Solvent Concentration	
	CF/DMF Group	AA/CF Group
2 wt%	1:1 CF/DMF	1:1 AA/CF
	3:1 CF/DMF	3:1 AA/CF
	100% CF	100% AA
4 wt%	1:1 CF/DMF	1:1 AA/CF
	3:1 CF/DMF	3:1 AA/CF
	100% CF	100% AA
6 wt%	1:1 CF/DMF	1:1 AA/CF
	3:1 CF/DMF	3:1 AA/CF
	100% CF	100% AA

**Table 2:** Mean diameters of the particles (in µm) and standard deviations for all combinations of PCL concentration and solvent ratios based on SEM micrographs.

Mean Particle Diameter (µm)—SEM Analysis						
	1:1 CF/DMF	3:1 CF/DMF	100% CF	1:1 AA/CF	3:1 AA/CF	100% AA
2 wt%	0.92 ± 0.31	2.49 ± 1.41	9.90 ± 3.30	3.26 ± 1.07	2.65 ± 0.95	2.18 ± 0.88
4 wt%	3.77 ± 2.14	3.72 ± 1.76	14.26 ± 5.93	2.54 ± 1.62	2.19 ± 1.55	2.54 ± 0.70
6 wt%	4.1 ± 1.76	5.46 ± 3.06	11.35 ± 4.68	9.34 ± 3.63	5.05 ± 3.21	3.91 ± 2.45

**Table 3:** Hydrodynamic diameters (in µm), polydispersity index (PDI), and zeta potential (mV) values of the particles for each sample.

Samples	HD (µm)	PDI	Zeta Potential (mV)
2 wt% 100% CF	16.2 ± 0.0	0.3	−0.06 ± 2.32
2 wt% 1:1 AA/CF	2.6 ± 1.0	0.05	−0.51 ± 2.31
2 wt% 3:1 AA/CF	6.9 ± 1.8	0.3	−0.79 ± 2.31
2 wt% 100% AA	7.9 ± 0.5	0.5	−0.49 ± 2.32
4 wt% 100% AA	3.8 ± 0.6	0.3	0.19 ± 2.32

## Data Availability

Data are available from the corresponding author upon reasonable request.
